# Supplementary material for: Activation of visual rhodopsin probed by single-shot transient IR spectroscopy
Source: Biophys J. 2025 Jun 28;125(10):2245–9. doi: 10.1016/j.bpj.2025.06.030 (PMC13351994; doi:10.1016/j.bpj.2025.06.030)
Supplement: Document S1. Figures S1–S3 and supporting materials and methods [file mmc1.pdf]

Biophysical Journal, Volume *125*

Supplemental information

Activation of visual rhodopsin probed by single-shot transient IR spectroscopy

Luiz Schubert, Franz Bartl, and Joachim Heberle

Supplementary Information to: Activation of Visual Rhodopsin Probed by Single-Shot Transient IR Spectroscopy

Luiz Schubert[1], Franz Bartl[2], Joachim Heberle[1]*

[1]Experimental Molecular Biophysics, Department of Physics, Freie Universität Berlin, 14195 Berlin, Germany.

[2]Biophysical Chemistry, Institute for Biology, Humboldt-Universität zu Berlin, 10115 Berlin, Germany.

Material and Methods

Sample preparation

Rhodopsin (rod outer segments, ROS) samples were essentially prepared as described in [1]. ROS were dispersed in a buffer containing 20 mM 1,3-bis(tris(hydroxymethyl)methylamino)propane (BTP), 130 mM NaCl and 5 mM $MgCl_2$ in 100 % H_2O. For exchanging to D_2O, samples were repetitively washed with the same buffer prepared in D_2O. The procedure for the preparation of the transmission sample holder is essentially described in[2]. The membrane fragments were collected from the aqueous solution by centrifugation. The supernatant was removed, and the highly concentrated membrane-slurry was spread onto a CaF2 or BaF2 window using a spatula. The sample chamber was sealed by a second window separated by a spacer. Depending on the preparation and spectral range, the experiments were carried out using spacers with 6, 13, 25 or 50 μm thickness. The exact concentration of the samples has not been determined, but is likely to be in the lower mM range[2].

All time-resolved experiments were conducted at room temperature (T = 20-23°C) at pH/pD ~ 6. For trapping the Meta-I intermediate, ROS were dispersed in the aforementioned buffer at pD 9 and the experiment was conducted at T = 5°C. Sample preparation and all experiments were conducted under dim red light conditions.

IR spectroscopy

Steady-state FTIR spectra were recorded using a Bruker Vertex 80V FTIR spectrometer at a spectral resolution of 4 cm^{-1}. Light activation of rhodopsin was achieved using continuous illumination with a 530 nm LED. The setup using the EC-QCL is essentially described in [3, 4]. The linearly spaced datapoints were logarithmically averaged to 60 datapoints per decade. DCS experiments were conducted using the commercially available spectrometer IRis-F1 from IRsweep, Stäfa, Switzerland [4, 5]. A recent hardware

update extends the accessible time range 32 to 128 ms. The linearly DCS data has been logarithmically averaged to 20 datapoints per decade. In both QCL-based experiments, pulsed light activation was done by a Nd:YAG laser (Minilite II, Continuum) with emission wavelength λ=532 nm and an energy density of E_{exc}/A = 4.5 mJ/cm^{-2}. Data analysis has been performed using a Python script as described in [4].

Supporting Information

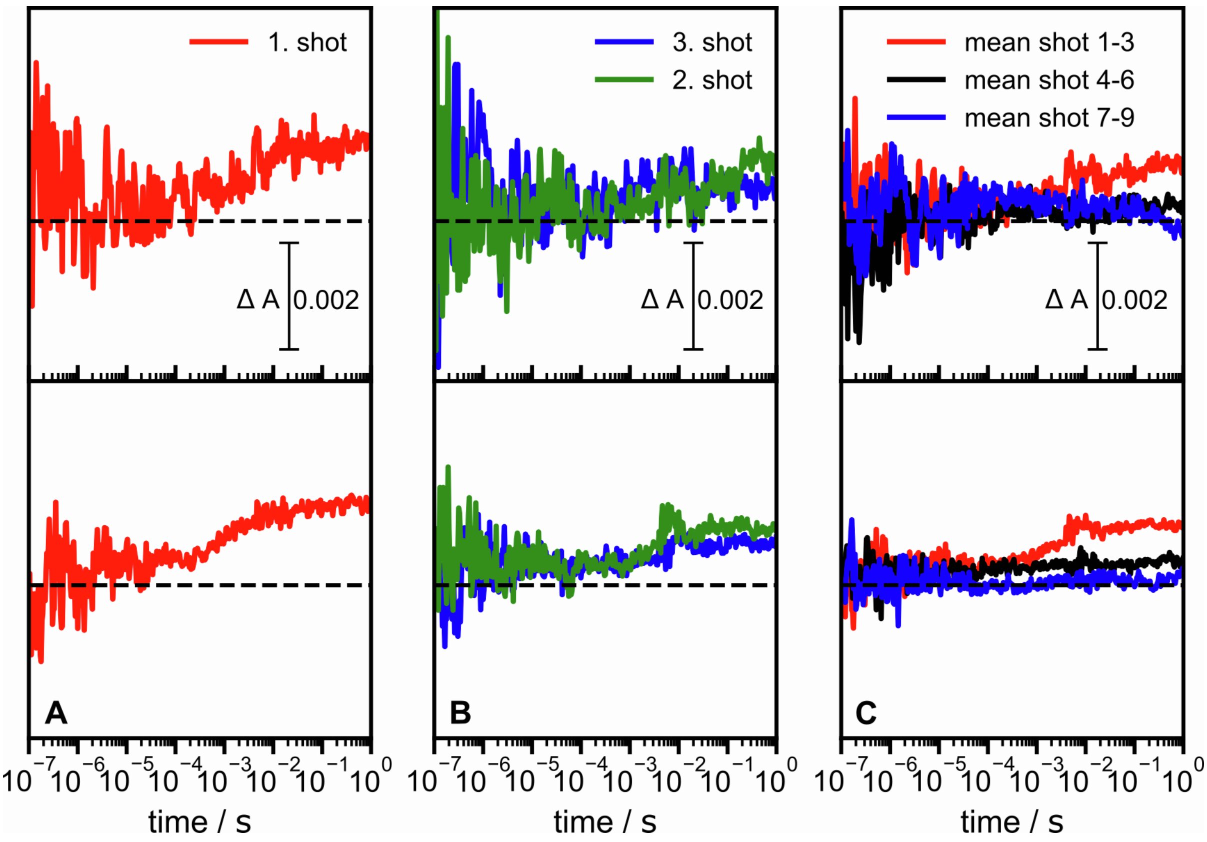

Figure S1: Photobleaching and acquisition averaging of multiple single-shot transients recorded on the same sample at 1644 cm^{-1}. Upper panel: Experiments conducted in H_2O at an optical pathlength of the sample of 13 µm. Lower panel: Experiments conducted in D_2O at an optical pathlength of the sample of 25 µm. A) Kinetic traces recorded after exposing the sample to the first laser excitation after preparation in the dark. B) Kinetic traces recorded after exposing the same sample to the second (green) and third (blue) laser flash. C) Kinetic traces obtained by co-averaging acquisitions 1-3 (red), 4-6 (black) and 7-9 (blue). The time between excitations was set to 8 s.

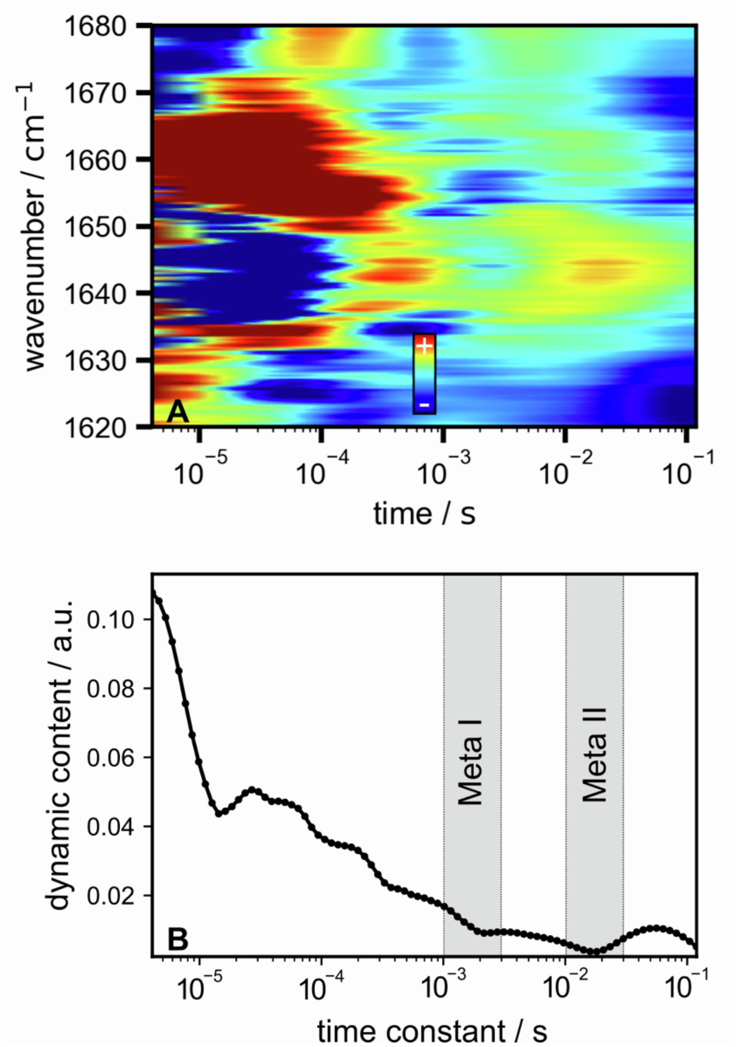

Figure S2: Dual-comb spectroscopy applied to the study of the rhodopsin photocycle. A) Heatmap of the light-induced absorption changes as a function of time and wavenumber. The plot shows LDA-fitted data. Note that the color code is scaled to properly display the data in the millisecond time range. Therefore, the large noise in the µs range is out of scale. B) Dynamic content (see ref.[6] for a description) obtained by lifetime density analysis (LDA). Dashed boxes indicate representative times where the Meta I and Meta II intermediates have been extracted. In order to obtain an appropriate modelling by the LDA, time constants larger than the measurement range were allowed (up to 1 s).

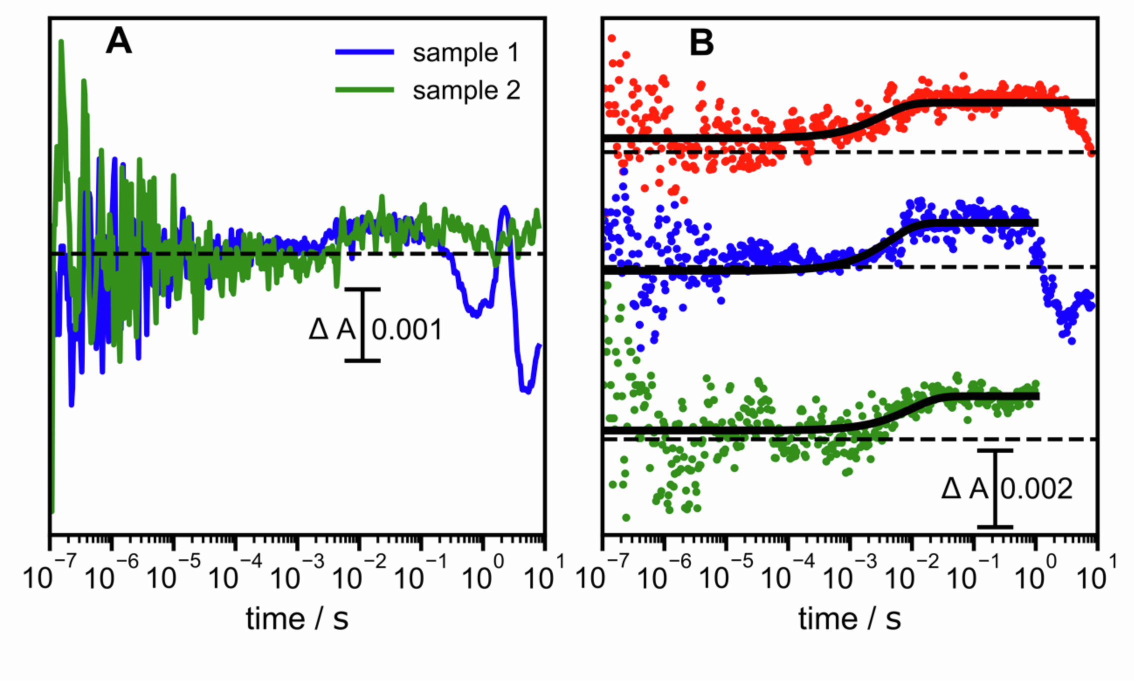

Figure S3: A) Single-shot transients recorded at 1741 cm^{-1} of two individual samples. The average of the two transients is shown in Figure 3 in the main text. As described in the main text, the drift on the timescale after 100 ms is only observed in one of the two transients and is attributed to an instability in the emission of the QCL in this experiment. B) Similar effects are observed in the amide I region at 1644 cm^{-1}. Therefore, the transients in the main text are shown until 1s after photoexcitation.

References

1. Ritter, E.; Zimmermann, K.; Heck, M.; Hofmann, K. P.; Bartl, F. J., Transition of Rhodopsin into the Active Metarhodopsin II State Opens a New Light-induced Pathway Linked to Schiff Base Isomerization*. *J. Biol. Chem.* **2004,** *279,* 48102-48111.
2. Bartl, F.; Ritter, E.; Hofmann, K. P., FTIR spectroscopy of complexes formed between metarhodopsin II and C-terminal peptides from the G-protein α- and γ-subunits. *FEBS Lett.* **2000,** *473,* 259-264.
3. Schultz, B. J.; Mohrmann, H.; Lorenz-Fonfria, V. A.; Heberle, J., Protein dynamics observed by tunable mid-IR quantum cascade lasers across the time range from 10 ns to 1 s. *Spectrochim. Acta, Pt. A: Mol. Biomol. Spectrosc,* **2018,** *188,* 666-674.
4. Schubert, L.; Langner, P.; Ehrenberg, D.; Lorenz-Fonfria, V. A.; Heberle, J., Protein conformational changes and protonation dynamics probed by a single shot using quantum-cascade-laser-based IR spectroscopy. *J. Chem. Phys.* **2022,** *156,* 204201.
5. Klocke, J. L.; Mangold, M.; Allmendinger, P.; Hugi, A.; Geiser, M.; Jouy, P.; Faist, J.; Kottke, T., Single-Shot Sub-microsecond Mid-infrared Spectroscopy on Protein Reactions with Quantum Cascade Laser Frequency Combs. *Anal. Chem.* **2018,** *90,* 10494-10500.
6. Ruf, J.; Bindschedler, F.; Buhrke, D., The molecular mechanism of light-induced bond formation and breakage in the cyanobacteriochrome TePixJ. *Phys. Chem. Chem. Phys.* **2023,** *25,* 6016-6024.
