# Supplementary material for: Activation of visual rhodopsin probed by single-shot transient IR spectroscopy
Source: Biophys J. 2025 Jun 28;125(10):2245–9. doi: 10.1016/j.bpj.2025.06.030 (PMC13351994; doi:10.1016/j.bpj.2025.06.030)
Supplement: Document S2. Article plus supporting material [file mmc2.pdf]

Biophysical Journal
Biophysical letter

Biophysical Society

Activation of visual rhodopsin probed by single-shot transient IR spectroscopy

Luiz Schubert,[1] Franz Bartl,[2] and Joachim Heberle[1,*]
[1]Experimental Molecular Biophysics, Department of Physics, Freie Universität Berlin, 14195 Berlin, Germany and [2]Biophysical Chemistry, Institute for Biology, Humboldt-Universität zu Berlin, 10115 Berlin, Germany

ABSTRACT In order to understand the structure-function relationships of proteins, it is important to study their dynamics under physiological conditions. The advent of X-ray free electron lasers has made it possible to obtain the three-dimensional structures of proteins and their reaction intermediates at room temperature. However, these experiments are very demanding and require extensive planning. Here, we demonstrate that time-resolved infrared difference spectroscopy using quantum cascade lasers is a powerful tool for studying the dynamics of protein conformational changes. This method complements structural biology experiments and aids in data interpretation. We demonstrate the feasibility of studying the irreversible photoreaction of the G-protein-coupled receptor rhodopsin using single shots.

SIGNIFICANCE Reaction-induced infrared difference spectroscopy is a well-established tool for gaining insight into the functional mechanism of proteins. Its chemical sensitivity provides insight into almost all molecular entities involved in the reaction under study. Despite a few exceptions, infrared difference spectroscopy has long been limited to the study of light-driven and strictly reversible systems. The novel techniques presented here may provide an opportunity to overcome this limitation and open the field to studies of irreversible photoreactions. Together with the use of caged compounds or rapid mixing, the study of intrinsically light-insensitive reaction dynamics, such as the activation of ligand-activated G-protein-coupled receptors, may be considered in future studies.

Protein function is inherently linked to the protein's structure and the dynamical changes thereof. Serial crystallography allows the determination of functionally relevant structural changes by recording structural snapshots during the protein's reaction mechanism (1). Often, a priori knowledge of the reaction mechanism at study is required to select appropriate time delays. In this respect, time-resolved UV-visible spectroscopy has been instrumental in providing information on the reaction dynamics (2) but is intrinsically limited to chromoproteins. Instead, transient infrared (IR) spectroscopy (3) provides equivalent data on the reaction dynamics but, at the same time, complements the interpretation of structural data by providing detailed information on structural alterations of the protein on the level of single bonds (4,5). While serial crystallography using X-ray free electron lasers is a single-shot technique per se (the crystals get destroyed with each exposure) and can, therefore, be readily applied to the study of nonrepetitive protein reactions (6), transient IR spectroscopy is, nowadays, mostly restricted to the study of reversible (and intrinsically light-driven) systems where the same reaction can be probed repetitively using the same sample (7).

Recently, we benchmarked two table-top mid-IR spectrometers based on quantum cascade lasers (QCLs) by probing a reversible protein reaction using a single shot, mimicking the ability to probe irreversible reactions (8). In the present work, we applied these two techniques to the study of protein conformational dynamics associated with the irreversible activation of visual rhodopsin, which has been studied in great detail and which serves as a model system for G-protein-coupled receptors (for a detailed review, see (9)).

Visual rhodopsin, a G-protein coupled receptor, undergoes a series of thermally activated reactions after the initial photoisomerization from 11-*cis* to all-*trans* retinal. While photoisomerization occurs on a fs-ps timescale, the thermally activated reactions leading to the signaling state Meta II, where G-protein binding occurs, take place up to 10 orders of magnitude later (Fig. 1 *A*) (9). Meta II state formation is associated with distinct structural changes of the protein,

Submitted April 16, 2025, and accepted for publication June 23, 2025.
*Correspondence: joachim.heberle@fu-berlin.de
Editor: Ana Nicoleta Bondar.
https://doi.org/10.1016/j.bpj.2025.06.030
© 2025 The Author(s). Published by Elsevier Inc. on behalf of Biophysical Society.
This is an open access article under the CC BY license (http://creativecommons.org/licenses/by/4.0/).

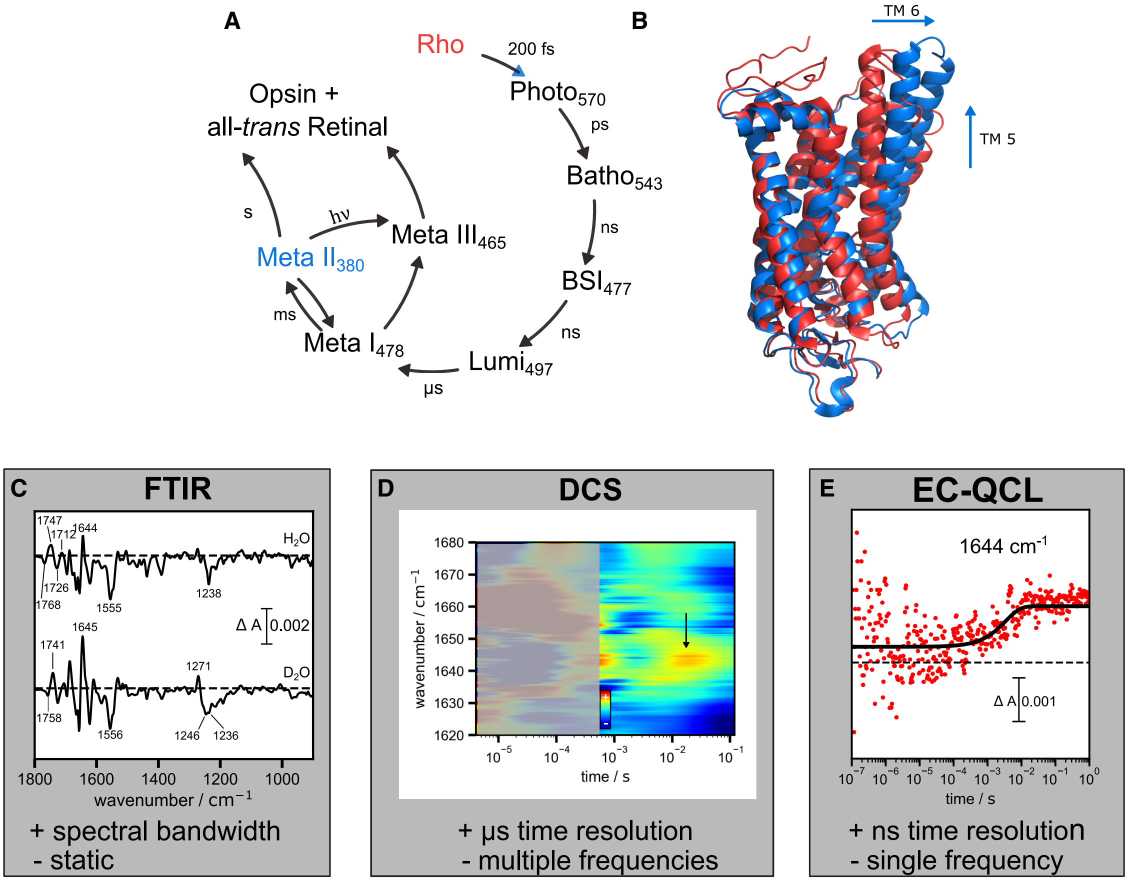

FIGURE 1 IR spectroscopic approaches for the study of the activation of the G-protein-coupled receptor rhodopsin. (*A*) Photocycle of bovine rhodopsin adapted from (9). (*B*) Crystal structures of bovine rhodopsin (*red*, PDB: 1U19) and the Meta II intermediate (*blue*, PDB: 3PXO) adapted from (14). Arrows indicate the most prominent movements of the helices upon receptor activation. (*C–E*) Exemplary datasets obtained in this study. (*C*) Static spectra recorded by FTIR spectroscopy in H_2O (*top*) and D_2O (*bottom*). (*D*) Spectro-temporal data obtained by dual-comb spectroscopy (LDA-fitted data). The scaling of the contour plot was adjusted in order to highlight the positive absorption feature around 1644 cm^{-1}, highlighted with the arrow. Therefore, the large noise in the dataset on the sub-ms timescale is out of scale (*gray shading*). (*E*) Single-shot transient obtained by EC-QCL-based spectroscopy. Red datapoints show raw data, while the black trace shows a monoexponential fit.

such as deprotonation of the Schiff base, subsequent proton transfer reactions, and movement of helices 5 and 6 (Fig. 1 *B*) (10). Unlike bacterial rhodopsins, the photoreaction in bovine rhodopsin is not reversible, as the active Meta II state decays into opsin, and all-*trans* retinal and the inactive 11-*cis*-bound ground state can only be regenerated by a complex cellular metabolism. Photoregeneration of the active Meta II failed to restore the ground state, leading to the formation of Meta III instead, a product with different properties (Fig. 1) (11). Hence, a spectroscopic technique that records protein conformational changes on the ns-to-s timescale is desired to study the dynamics of receptor activation. Among other biophysical techniques, such as electron paramagnetic resonance, fluorescence resonance energy transfer, NMR spectroscopy, and X-ray scattering techniques, mid-IR spectroscopy has the advantage of being both label free and nondestructive. However, a combination of different experiments is required to obtain sufficient spectral and dynamic information (Fig. 1, *C–E*). Conventional Fourier transform IR spectroscopy (FTIR spectroscopy) has been used to obtain static spectra of intermediates covering a broad spectral range with information on a multitude of molecular vibrations. By choosing appropriate conditions (e.g., pH, water content, and temperature), difference spectra can be recorded where pure intermediates or mixtures of intermediates are trapped (12). Fig. 1 *C* shows the difference spectrum of the rhodopsin-to-Meta II transition recorded at a pH/pD of ~6 and room temperature. In addition to the Meta II marker band at 1644 cm^{-1}, which has been assigned to an amide I vibration indicative of protein conformational changes, the spectrum provides useful information on the configuration of the retinal chromophore (~1500 and 1200 cm^{-1}), the protonation state and environment of amino acid side chains with terminal carboxylic acids (1800–1700 cm^{-1}), and other residues (12,13). The step-scan FTIR spectroscopy technique has been applied to microbial rhodopsins for studying the reaction dynamics on an ns-to-s timescale but requires many repetitions to achieve a sufficient signal/noise ratio (SNR). Therefore, it

is not applicable to vertebrate rhodopsin due to its noncyclic photoreaction unless the sample is exchanged for a fresh one after each shot. In contrast, QCL-based methods are limited to a comparably narrow spectral range. However, partly because of the QCL's higher emission power as compared to conventional mid-IR light sources, these techniques provide information about the dynamics of the photoreaction with sufficient SNRs averaging only a few acquisitions. Dual-comb spectroscopy (DCS) provides spectro-temporal data in the range between 1680 and 1620 cm^{-1}, covering a time range from 10^{-6} to 10^{-1} s (Fig. 1 *D*) in a single acquisition. Instead, the setup employing an external cavity QCL (EC-QCL) provides a single transient of the band at 1644 cm^{-1}, albeit with higher time resolution (Fig. 1 *E*). We demonstrate that both QCL-based mid-IR absorption techniques are well suited to study the dynamics of irreversible (photo)reactions *after* the reaction at study has been spectrally characterized by a preceding FTIR spectroscopy experiment.

Both QCL-based methods are capable of studying the dynamics of receptor activation at room temperature in solution (Fig. 2), but each has distinct advantages and disadvantages. In contrast to the EC-QCL setup, the emitted optical power in DCS is distributed over several laser lines (see (8) for a more technical discussion), which imposes stronger limitations on the optical pathlength of the sample as compared to the EC-QCL. This becomes particularly relevant when measuring aqueous samples, where the water bending mode overlaps with the amide I band. On the other hand, longer optical pathlengths lead to stronger difference signals, as more molecules are probed. Therefore, one must compromise between high transmittance and strong difference signals to optimize SNRs. Here, the DCS experiments were performed on rod outer segment fragments dispersed in a deuterated buffer using a 25 μm spacer, while the EC-QCL experiments were done in an aqueous solution using 13 and 6 μm spacers. While all three EC-QCL transients (Fig. 2 *C*) were recorded by a single shot, the DCS dataset (Fig. 2, *A* and *B*) shows the average of nine single shots (three shots each on three samples). Averaging a few shots on a single sample is justified, as the visible laser excitation does not excite all molecules in the focal spot (cf. Fig. S1). DCS spectra were extracted at characteristic times for the Meta I and Meta II intermediates (Fig. 2, *A* and *B*; cf. Fig. S2). The Meta II spectrum agrees well with the spectrum obtained by FTIR spectroscopy trapping experiments recorded here and previously published ones (15,16). Characteristic bands at 1624 (−), 1644 (+), 1654 (−), and 1670 (−) cm^{-1} identify the appearance of the Meta II state. The small shoulder at 1634 cm^{-1} resolved in FTIR spectroscopy experiments at 2 cm^{-1} resolution is not resolved in the DCS experiments after smoothing to 8.1 cm^{-1}. The Meta I intermediate spectrum has a lower SNR, but the bands at 1625 and 1655 cm^{-1} are resolved (Fig. 2 *A*). The smaller positive bands at 1632, 1643, and 1663 cm^{-1} observable in the FTIR spectroscopy spectrum, which have also been reported in literature (16,17), are not resolved in the spectrum obtained by DCS. Kinetic traces extracted from the spectro-temporal DCS dataset are comparably noisy and are therefore not shown. The transients recorded by the EC-QCL setup show higher SNRs and are highly reproducible. Fig. 2 *C* shows three transients recorded at 1644 cm^{-1} on three individually prepared samples. All transients show a rise of the band in the ms time range, well above the noise level. Monoexponential fits yield time constants of 3.4, 4.4, and 9.7 ms for each of the three samples.

Despite the lower SNR below 1 ms, the EC-QCL setup and DCS are able to probe the dynamics of Meta II formation involving protein conformational changes occurring on the millisecond timescale. Our results are in good agreement with time-resolved electron paramagnetic resonance experiments, where the outward tilt of helix 6 is observed with a time constant of 1.9 ms in detergent-solubilized rhodopsin (18).

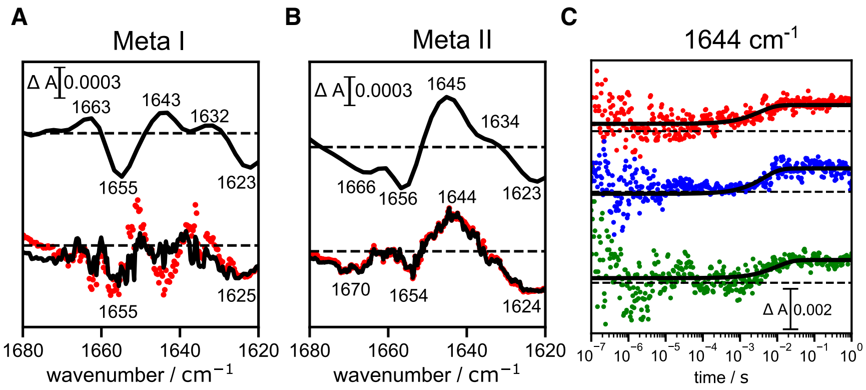

FIGURE 2 Protein conformational changes associated with receptor activation studied by QCL-based spectroscopy. (*A* and *B*) Meta I (*A*) and Meta II (*B*) spectra obtained by DCS (*bottom*) in comparison to Meta I and Meta II spectra obtained by FTIR spectroscopy (*top*) in D_2O. The DCS spectra display raw data in red the results from lifetime density analysis (LDA) in black. (*C*) Single-frequency transients recorded at 1644 cm^{-1} in H_2O with the EC-QCL setup. Each trace shows data obtained from the first single shot applied to a freshly prepared sample. To facilitate comparison, the blue and green traces are multiplied by factors of 1.5 and 1.8, respectively, to match the intensity of the red trace. The different amplitudes are due to the use of different spacers (*red*: 13 μm; *blue* and *green*: 6 μm). Dots display raw data points of three individual measurements, and black lines represent monoexponential fits. Note that the red trace in (*C*) shows the same data as already shown in Fig. 1 *E*.

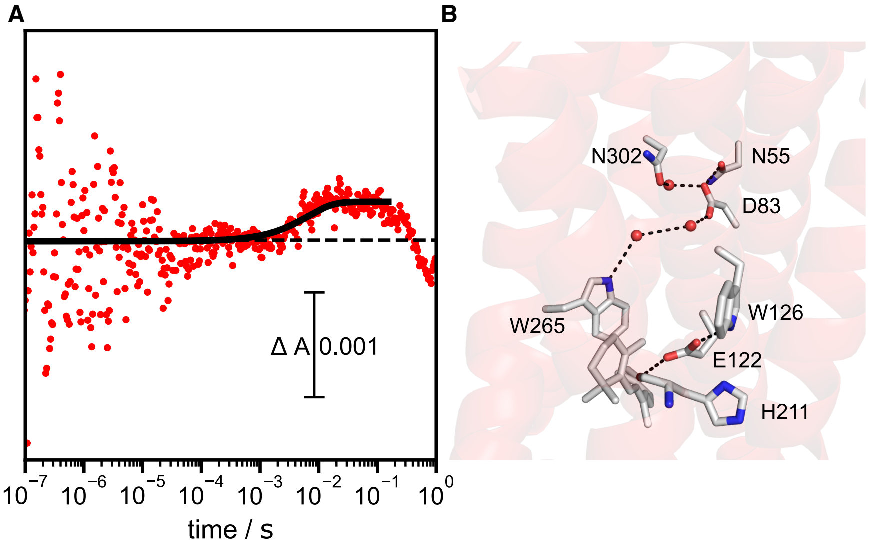

FIGURE 3 Dynamics of environmental changes of single amino acids resolved by single-frequency IR spectroscopy. (*A*) Transient recorded at 1741 cm^{-1} by the EC-QCL setup. The data shown are the average of two individual single-shot experiments recorded in D_2O using a 50 μm spacer. Raw data are shown in red, with a monoexponential fit shown in black. The absorption changes monitored at 1741 cm^{-1} are associated with environmental changes of D83 and E122. (*B*) Environments of D83 and E122 (PDB: 1U19). Water molecules are shown as red spheres.

Reaction-induced difference spectroscopy is particularly useful to monitor not just conformational changes of the protein backbone but also perturbations of single amino acids and protonation states of protonatable amino residues (3). The C=O stretching vibrational bands of amino acids with terminal carboxylic acids appear between 1800 and 1700 cm^{-1}. The FTIR spectroscopy difference spectrum (Fig. 1 *A*) shows several bands in this region with a prominent feature at 1768 (−), 1747 (+), and 1726 cm^{-1} (−), which redshifts upon deuteration. This feature has been assigned to a change in the environment of protonated D83 and E122 (Fig. 3 *B* and 12,19). Notably, in this spectral region, another, albeit smaller, band contributes to the difference signal, which has been assigned to the carboxylic acid ester vibration of surrounding lipid molecules (20). The substantially smaller band at 1712 cm^{-1} (+) in H_2O (~1705 cm^{-1} in D_2O) has been assigned to the protonation of the primary counterion E113 (12). To illustrate that QCL-based methods are capable of probing these minute structural changes of single amino acids, the transient of the band occurring at 1741 cm^{-1} is monitored in D_2O (Fig. 3 *A*). The absorption increases in the ms time range to less than 5×10^{-4}, which is still well above the noise level. The rise of this band (τ = 6.5 ms) coincides with the rise of the band at 1644 cm^{-1} and hence Meta II formation under the present conditions (~23°C, pD = 6). The apparent decay of the band at >200 ms is observed in only one of the two transients and is likewise observed in some transients of the amide I region (cf. Fig. S3). It is caused by very slow fluctuations in the emission of QCLs and is, thus, not a response from the sample. The inclusion of a second detector as a reference (21) in our setup will help to deal with this artifact.

Mid-IR difference spectroscopy is a powerful tool to study protein reactions, in particular in concord with (time-resolved) protein crystallographic experiments (4,5). A thorough spectral characterization by steady-state FTIR spectroscopy in combination with time-resolved techniques based on table-top QCL spectrometers provides valuable insights into the mechanism and dynamics of the reaction at study. The high SNR achievable by the QCL-based techniques presented here allows single-shot experiments to be performed and thus information on the dynamics of an irreversible reaction to be obtained, which is not possible with conventional time-resolved FTIR spectroscopy techniques. Studying the dynamics of the amide I vibration reporting on backbone changes of the proteins aids serial crystallography experiments by selecting appropriate pump-probe delays. Additionally, providing information on protonation states of certain residues provides information often not accessible by structural methods. Undoubtedly, pump-probe methods with higher time resolution are capable of collecting data with high SNRs, too. If diffusion-controlled reactions between the enzyme and substrate are the scope, high time resolution (sub-ns) is usually not required. Instead, it is important to extend the measurable timescales toward milliseconds and beyond. This has already been achieved by synchronizing two pulsed laser systems (22,23). Other approaches, e.g., employing a dispersive spectrometer in conjunction with a very brilliant light source at a synchrotron, are also promising techniques but comparably costly and time consuming to set up (14). Instead, table-top spectrometers employing QCLs are compact and relatively simple alternatives from the users' point of view. We therefore believe that QCL-based mid-IR spectrometers are promising tools for future studies of the dynamics of biological systems. To achieve this goal, we propose implementing such time-resolved techniques at current free electron laser and synchrotron beamlines to correlate transient changes in

electron density as determined by X-ray crystallography with vibrational changes as recorded by IR spectroscopy on the same sample.

ACKNOWLEDGMENTS

We thank Pit Langner for excellent technical assistance regarding the QCL-based spectrometers and Martin Heck for the preparation of rhodopsin samples. This work was funded by the German Research Foundation (DFG) via SFB1078 to J.H. (project B3) and F.B. (project B5). This paper is dedicated to Klaus-Peter Hofmann, who passed away on July 20, 2024. Peter was a pioneer in studying the molecular mechanism of visual rhodopsin and the downstream signaling cascade. He was a mentor and role model for us.

AUTHOR CONTRIBUTIONS

L.S. conducted the experiments, analyzed the data, and wrote the first draft of the manuscript. F.B. (and co-workers) prepared the samples and joined L.S. for the experiments. J.H. initiated and coordinated the project. F.B. and J.H. revised the manuscript.

DECLARATION OF INTERESTS

The authors declare no competing financial interests.

SUPPORTING MATERIAL

Supporting material can be found online at https://doi.org/10.1016/j.bpj.2025.06.030.

REFERENCES

1. Standfuss, J. 2019. Membrane protein dynamics studied by X-ray lasers – or why only time will tell. *Curr. Opin. Struct. Biol.* 57:63–71.
2. Nogly, P., T. Weinert, …, J. Standfuss. 2018. Retinal isomerization in bacteriorhodopsin captured by a femtosecond x-ray laser. *Science.* 361:eaat0094.
3. Radu, I., M. Schleeger, …, J. Heberle. 2009. Time-resolved methods in biophysics. 10. Time-resolved FT-IR difference spectroscopy and the application to membrane proteins. *Photochem. Photobiol. Sci.* 8:1517–1528.
4. Skopintsev, P., D. Ehrenberg, …, J. Standfuss. 2020. Femtosecond-to-millisecond structural changes in a light-driven sodium pump. *Nature.* 583:314–318.
5. Mous, S., G. Gotthard, …, P. Nogly. 2022. Dynamics and mechanism of a light-driven chloride pump. *Science.* 375:845–851.
6. Gruhl, T., T. Weinert, …, V. Panneels. 2023. Ultrafast structural changes direct the first molecular events of vision. *Nature.* 615:939–944.
7. Lorenz-Fonfria, V. A. 2020. Infrared Difference Spectroscopy of Proteins: From Bands to Bonds. *Chem. Rev.* 120:3466–3576.
8. Schubert, L., P. Langner, …, J. Heberle. 2022. Protein conformational changes and protonation dynamics probed by a single shot using quantum-cascade-laser-based IR spectroscopy. *J. Chem. Phys.* 156:204201.
9. Ernst, O. P., D. T. Lodowski, …, H. Kandori. 2014. Microbial and animal rhodopsins: Structures, functions, and molecular mechanisms. *Chem. Rev.* 114:126–163.
10. Choe, H.-W., Y. J. Kim, …, O. P. Ernst. 2011. Crystal structure of metarhodopsin II. *Nature.* 471:651–655.
11. Bartl, F. J., E. Ritter, and K. P. Hofmann. 2001. Signaling States of Rhodopsin: ABSORPTION OF LIGHT IN ACTIVE METARHODOPSIN II GENERATES AN ALL-*TRANS* RETINAL BOUND INACTIVE STATE. *J. Biol. Chem.* 276:30161–30166.
12. Siebert, F. 1995. Application of FTIR Spectroscopy to the Investigation of Dark Structures and Photoreactions of Visual Pigments. *Isr. J. Chem.* 35:309–323.
13. Zaitseva, E., M. F. Brown, and R. Vogel. 2010. Sequential Rearrangement of Interhelical Networks Upon Rhodopsin Activation in Membranes: The Meta IIa Conformational Substate. *J. Am. Chem. Soc.* 132:4815–4821.
14. Ritter, E., L. Puskar, …, U. Schade. 2019. Féry Infrared Spectrometer for Single-Shot Analysis of Protein Dynamics. *J. Phys. Chem. Lett.* 10:7672–7677.
15. Siebert, F., W. Mäntele, and K. Gerwert. 1983. Fourier-transform infrared spectroscopy applied to rhodopsin. *Eur. J. Biochem.* 136:119–127.
16. Ganter, U. M., C. Longstaff, …, F. Siebert. 1991. Fourier transform infrared studies of active-site-methylated rhodopsin. Implications for chromophore-protein interaction, transducin activation, and the reaction pathway. *Biophys. J.* 59:640–644.
17. Vogel, R., F. Siebert, …, M. Sheves. 2005. Agonists and Partial Agonists of Rhodopsin: Retinals with Ring Modifications. *Biochemistry.* 44:11684–11699.
18. Knierim, B., K. P. Hofmann, …, W. L. Hubbell. 2007. Sequence of late molecular events in the activation of rhodopsin. *Proc. Natl. Acad. Sci. USA.* 104:20290–20295.
19. Fahmy, K., F. Jäger, …, F. Siebert. 1993. Protonation states of membrane-embedded carboxylic acid groups in rhodopsin and metarhodopsin II: a Fourier-transform infrared spectroscopy study of site-directed mutants. *Proc. Natl. Acad. Sci. USA.* 90:10206–10210.
20. Beck, M., F. Siebert, and T. P. Sakmar. 1998. Evidence for the specific interaction of a lipid molecule with rhodopsin which is altered in the transition to the active state metarhodopsin II. *FEBS Lett.* 436:304–308.
21. Dekmak, M. Y., S. M. Mäusle, …, H. Dau. 2024. Tracking the first electron transfer step at the donor side of oxygen-evolving photosystem II by time-resolved infrared spectroscopy. *Photosynth. Res.* 162:353–369.
22. Greetham, G. M., D. Sole, …, M. Towrie. 2012. Time-resolved multiple probe spectroscopy. *Rev. Sci. Instrum.* 83:103107.
23. Helbing, J., and P. Hamm. 2023. Versatile Femtosecond Laser Synchronization for Multiple-Timescale Transient Infrared Spectroscopy. *J. Phys. Chem. A.* 127:6347–6356.

Biophysical Journal, Volume *125*

Supplemental information

Activation of visual rhodopsin probed by single-shot transient IR spectroscopy

Luiz Schubert, Franz Bartl, and Joachim Heberle

Supplementary Information to: Activation of Visual Rhodopsin Probed by Single-Shot Transient IR Spectroscopy

Luiz Schubert[1], Franz Bartl[2], Joachim Heberle[1]*

[1]Experimental Molecular Biophysics, Department of Physics, Freie Universität Berlin, 14195 Berlin, Germany.

[2]Biophysical Chemistry, Institute for Biology, Humboldt-Universität zu Berlin, 10115 Berlin, Germany.

Material and Methods

Sample preparation

Rhodopsin (rod outer segments, ROS) samples were essentially prepared as described in [1]. ROS were dispersed in a buffer containing 20 mM 1,3-bis(tris(hydroxymethyl)methylamino)propane (BTP), 130 mM NaCl and 5 mM $MgCl_2$ in 100 % H_2O. For exchanging to D_2O, samples were repetitively washed with the same buffer prepared in D_2O. The procedure for the preparation of the transmission sample holder is essentially described in[2]. The membrane fragments were collected from the aqueous solution by centrifugation. The supernatant was removed, and the highly concentrated membrane-slurry was spread onto a CaF2 or BaF2 window using a spatula. The sample chamber was sealed by a second window separated by a spacer. Depending on the preparation and spectral range, the experiments were carried out using spacers with 6, 13, 25 or 50 µm thickness. The exact concentration of the samples has not been determined, but is likely to be in the lower mM range[2].

All time-resolved experiments were conducted at room temperature (T = 20-23°C) at pH/pD ~ 6. For trapping the Meta-I intermediate, ROS were dispersed in the aforementioned buffer at pD 9 and the experiment was conducted at T = 5°C. Sample preparation and all experiments were conducted under dim red light conditions.

IR spectroscopy

Steady-state FTIR spectra were recorded using a Bruker Vertex 80V FTIR spectrometer at a spectral resolution of 4 cm^{-1}. Light activation of rhodopsin was achieved using continuous illumination with a 530 nm LED. The setup using the EC-QCL is essentially described in [3, 4]. The linearly spaced datapoints were logarithmically averaged to 60 datapoints per decade. DCS experiments were conducted using the commercially available spectrometer IRis-F1 from IRsweep, Stäfa, Switzerland [4, 5]. A recent hardware

update extends the accessible time range 32 to 128 ms. The linearly DCS data has been logarithmically averaged to 20 datapoints per decade. In both QCL-based experiments, pulsed light activation was done by a Nd:YAG laser (Minilite II, Continuum) with emission wavelength λ=532 nm and an energy density of E_{exc}/A = 4.5 mJ/cm^{-2}. Data analysis has been performed using a Python script as described in [4].

Supporting Information

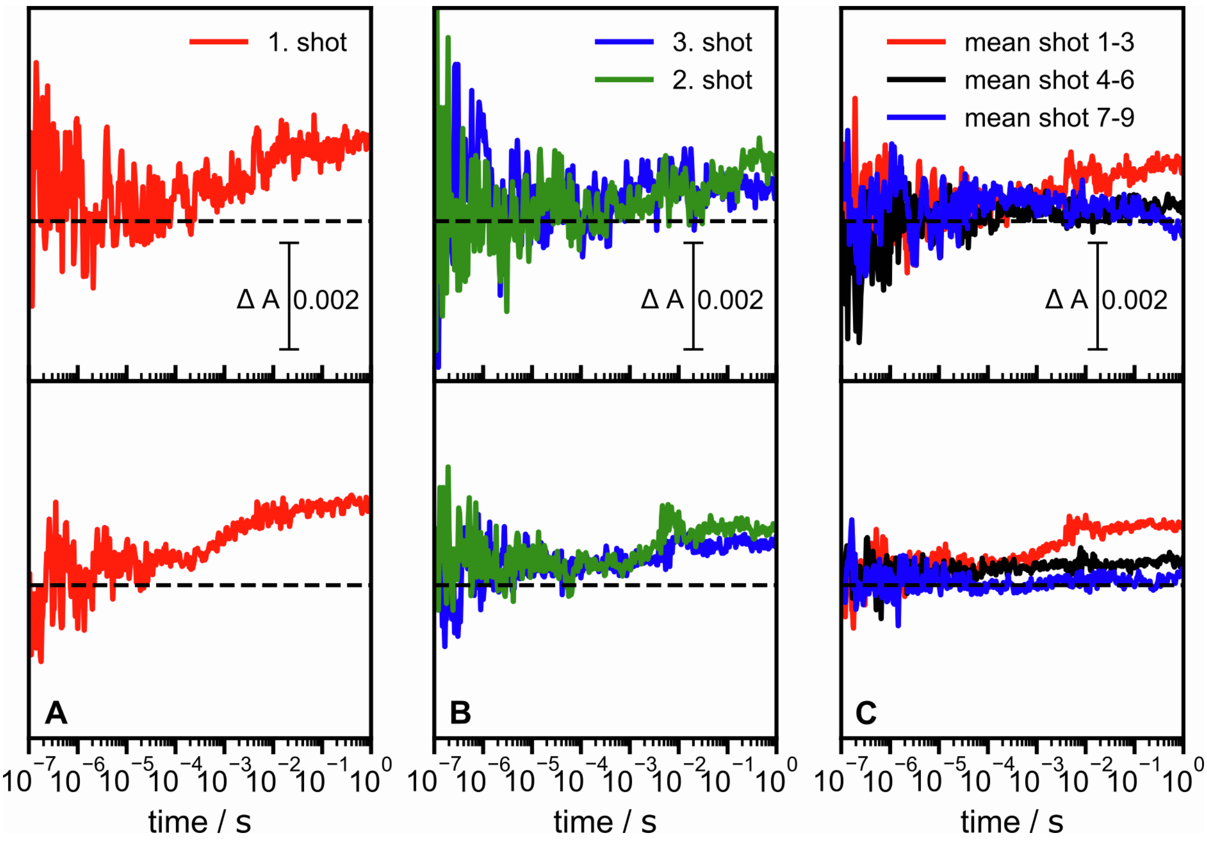

Figure S1: Photobleaching and acquisition averaging of multiple single-shot transients recorded on the same sample at 1644 cm^{-1}. Upper panel: Experiments conducted in H_2O at an optical pathlength of the sample of 13 µm. Lower panel: Experiments conducted in D_2O at an optical pathlength of the sample of 25 µm. A) Kinetic traces recorded after exposing the sample to the first laser excitation after preparation in the dark. B) Kinetic traces recorded after exposing the same sample to the second (green) and third (blue) laser flash. C) Kinetic traces obtained by co-averaging acquisitions 1-3 (red), 4-6 (black) and 7-9 (blue). The time between excitations was set to 8 s.

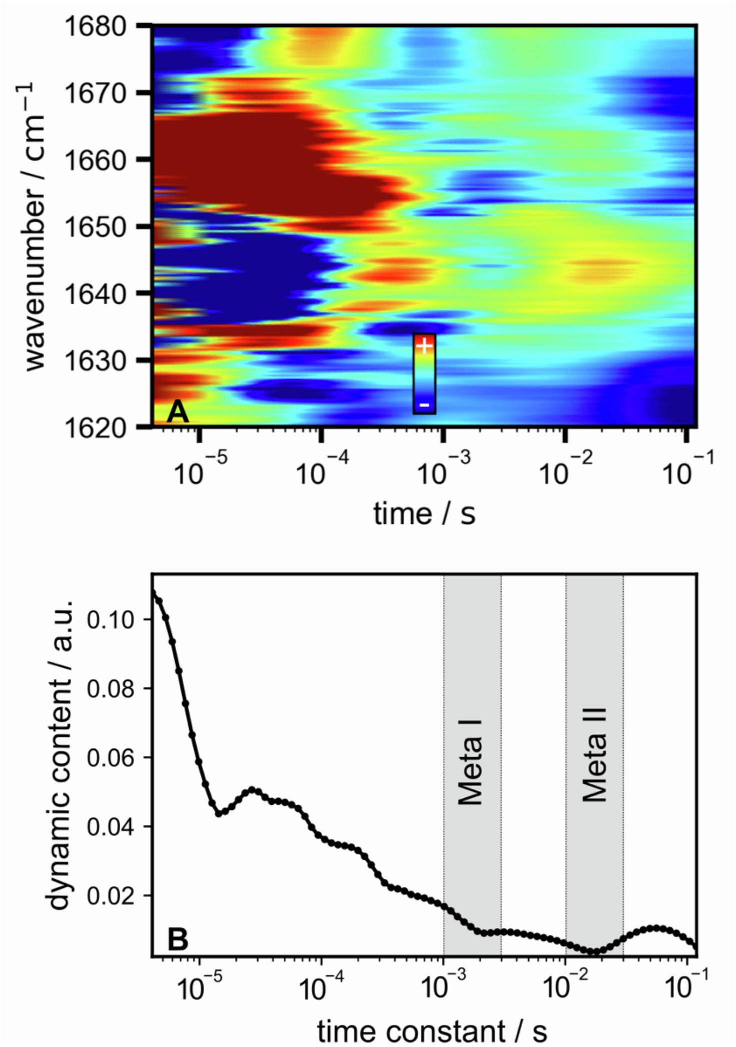

Figure S2: Dual-comb spectroscopy applied to the study of the rhodopsin photocycle. A) Heatmap of the light-induced absorption changes as a function of time and wavenumber. The plot shows LDA-fitted data. Note that the color code is scaled to properly display the data in the millisecond time range. Therefore, the large noise in the µs range is out of scale. B) Dynamic content (see ref.[6] for a description) obtained by lifetime density analysis (LDA). Dashed boxes indicate representative times where the Meta I and Meta II intermediates have been extracted. In order to obtain an appropriate modelling by the LDA, time constants larger than the measurement range were allowed (up to 1 s).

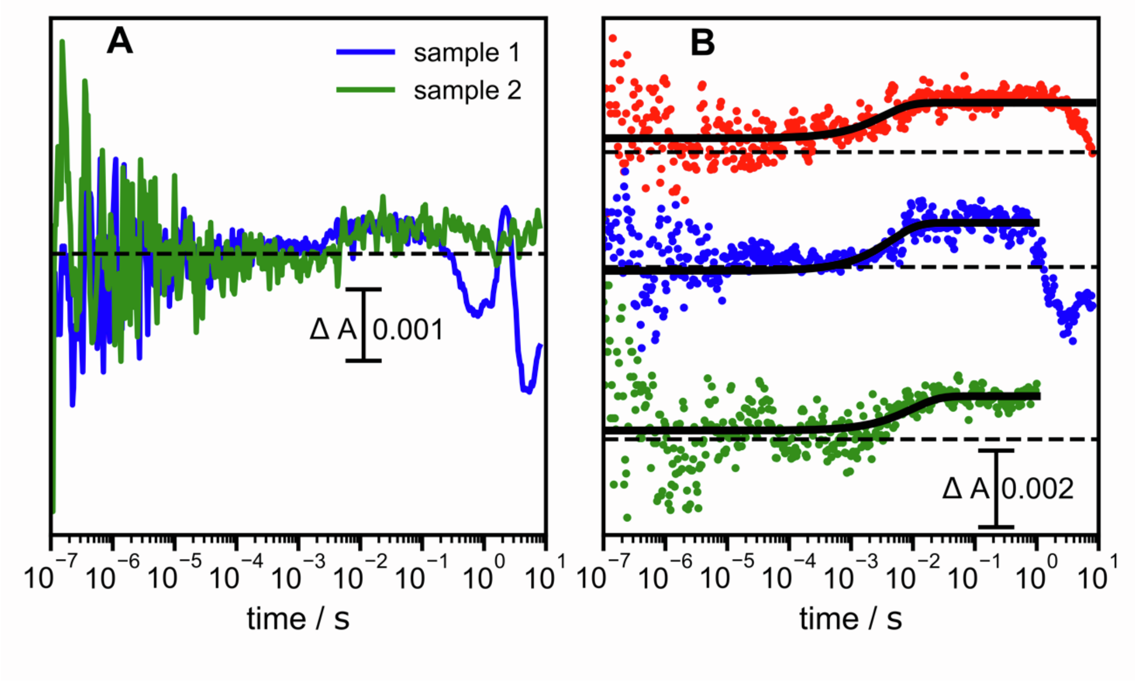

Figure S3: A) Single-shot transients recorded at 1741 cm^{-1} of two individual samples. The average of the two transients is shown in Figure 3 in the main text. As described in the main text, the drift on the timescale after 100 ms is only observed in one of the two transients and is attributed to an instability in the emission of the QCL in this experiment. B) Similar effects are observed in the amide I region at 1644 cm^{-1}. Therefore, the transients in the main text are shown until 1s after photoexcitation.

References

1. Ritter, E.; Zimmermann, K.; Heck, M.; Hofmann, K. P.; Bartl, F. J., Transition of Rhodopsin into the Active Metarhodopsin II State Opens a New Light-induced Pathway Linked to Schiff Base Isomerization*. *J. Biol. Chem.* **2004,** *279,* 48102-48111.
2. Bartl, F.; Ritter, E.; Hofmann, K. P., FTIR spectroscopy of complexes formed between metarhodopsin II and C-terminal peptides from the G-protein α- and γ-subunits. *FEBS Lett.* **2000,** *473,* 259-264.
3. Schultz, B. J.; Mohrmann, H.; Lorenz-Fonfria, V. A.; Heberle, J., Protein dynamics observed by tunable mid-IR quantum cascade lasers across the time range from 10 ns to 1 s. *Spectrochim. Acta, Pt. A: Mol. Biomol. Spectrosc,* **2018,** *188,* 666-674.
4. Schubert, L.; Langner, P.; Ehrenberg, D.; Lorenz-Fonfria, V. A.; Heberle, J., Protein conformational changes and protonation dynamics probed by a single shot using quantum-cascade-laser-based IR spectroscopy. *J. Chem. Phys.* **2022,** *156,* 204201.
5. Klocke, J. L.; Mangold, M.; Allmendinger, P.; Hugi, A.; Geiser, M.; Jouy, P.; Faist, J.; Kottke, T., Single-Shot Sub-microsecond Mid-infrared Spectroscopy on Protein Reactions with Quantum Cascade Laser Frequency Combs. *Anal. Chem.* **2018,** *90,* 10494-10500.
6. Ruf, J.; Bindschedler, F.; Buhrke, D., The molecular mechanism of light-induced bond formation and breakage in the cyanobacteriochrome TePixJ. *Phys. Chem. Chem. Phys.* **2023,** *25,* 6016-6024.
